# The Aromatase–Estrogen System in the Testes of Non-Mammalian Vertebrates

**DOI:** 10.3390/ani11061763

**Published:** 2021-06-12

**Authors:** Luigi Rosati, Sara Falvo, Gabriella Chieffi Baccari, Alessandra Santillo, Maria Maddalena Di Fiore

**Affiliations:** 1 Dipartimento di Biologia, Università degli Studi di Napoli “Federico II”, 80100 Napoli, Italy; luigi.rosati@unina.it; 2 Dipartimento di Scienze e Tecnologie Ambientali, Biologiche e Farmaceutiche, Università degli Studi della Campania “Luigi Vanvitelli”, 81100 Caserta, Italy; sara.falvo@unicampania.it (S.F.); gabriella.chieffi@unicampania.it (G.C.B.); alessandra.santillo@unicampania.it (A.S.)

**Keywords:** P450 aromatase, estradiol, spermatogenesis, steroidogenesis, reproduction, testis, amphibians, reptiles, birds, seasonal reproductive cycle

## Abstract

**Simple Summary:**

The aromatase–estrogen system plays a key role in gonadal sex differentiation in amphibians, reptiles, and birds during development. In adults of seasonal breeding species, aromatase activity and estrogen levels can act as an “on/off” switch for spermatogenesis and can also promote spermiogenesis.

**Abstract:**

Estrogens are important physiological regulators of testicular activity in vertebrates. Estrogen levels depend on the activity of P450 aromatase, the enzyme responsible for the irreversible conversion of testosterone into 17β-estradiol. Therefore, P450 aromatase is the key player in the aromatase–estrogen system. The present review offers a comparative overview of P450 aromatase activity in male gonads of amphibians, reptiles, and birds, with a particular emphasis on the functions of the aromatase–estrogen system in these organisms during their developmental and adult stages. The aromatase–estrogen system appears to be crucial for the sex differentiation of gonads in vertebrates. Administration of aromatase inhibitors prior to sexual differentiation of gonads results in the development of males rather than females. In adults, both aromatase and estrogen receptors are expressed in somatic cells, Leydig and Sertoli cells, as well as germ cells, with certain differences among different species. In seasonal breeding species, the aromatase–estrogen system serves as an “on/off” switch for spermatogenesis. In some amphibian and reptilian species, increased estrogen levels in post-reproductive testes are responsible for blocking spermatogenesis, whereas, in some species of birds, estrogens function synergistically with testosterone to promote spermatogenesis. Recent evidence indicates that the production of the aromatase enzyme in excessive amounts reduces the reproductive performance in avian species of commercial interest. The use of aromatase inhibitors to improve fertility has yielded suitable positive results. Therefore, it appears that the role of the aromatase–estrogen system in regulating the testicular activity differs not only among the different classes of vertebrates but also among different species within the same class.

## 1. Introduction

Estrogens have historically been considered the hormones responsible for regulating female reproduction, although findings from the last three decades have established that estrogens and their receptors also regulate male reproductive organs. In mammals, testes provide 20% of total estrogens, the rest being produced in adipose tissue, brain, skin, and bones [1,2,3,4]. Estrogens, besides having important functions in the male reproductive system, are also necessary for normal fertility. In several mammalian species, estrogens are involved in the spermatogenesis control, particularly in spermiogenesis, as well as in the control of spermatozoa maturation along the male genital tract [1,2,3,4]. Estrogens are present in extremely high levels in the semen of several species [5]. The presence of the nuclear receptors of estrogen, namely, estradiol receptors α (ERα) and β (ERβ), in the germ cells and along the spermatic ducts provides evidence of the importance of estrogen in testicular functions [1,2,3,6]. With respect to their localizations, it appears that ERβ is mainly involved in the control of spermatogenesis and spermiogenesis, whereas ERα is more relevant during the control of spermatozoa migration and maturation [3]. The levels of estrogen are controlled by P450 aromatase [2,3], which is an enzymatic complex localized in the endoplasmic reticulum. P450 aromatase is composed of two proteins: a ubiquitous NADPH-cytochrome P450 reductase and a cytochrome P450 aromatase that contains the steroid-binding site and is responsible for catalyzing the irreversible aromatization of testosterone (T) into 17β-estradiol (E_2_) [2]. Leydig cells, testicular germ cells, and epididymal sperm cells contain aromatase and synthesize estrogen [2]. This wide range of estrogen functions in the mammalian male reproductive system highlights the necessity to understand their role and the distribution of their receptors together with aromatase occurrence in non-mammalian vertebrates. It is well-known that non-mammalian vertebrates have served as useful models for endocrinology research on reproduction. In seasonal reproductive species, both androgens and estrogens are involved in gonadal regulation either during the developmental stages or the seasonal stages of the reproductive cycle in adults. Because the influence of androgens and estrogens on reproductive performance depends on the balance of their concentrations, the aromatase enzyme could be a key player in this regard [3,7,8,9]. Furthermore, P450 aromatase and estrogens play a fundamental role in gonadal sex differentiation in egg-laying vertebrates. Therefore, in the present review, the existing knowledge on the role of P450 aromatase in the regulation of male gonads in amphibians, reptiles, and birds is discussed, and the estrogen functions in relation to the aromatase among different species are compared. The events of reproduction in wild species are interesting and offer subjects for investigations that may have broad implications in environmental endocrinology of reproduction. The ready availability of all stages of development and the ease of their maintenance make them good candidates for future studies.

## 2. Amphibians

Amphibians are of great interest to the researchers working on phylogenetic vertebrate evolution as they were the first tetrapods to conquer the terrestrial environment and are recognized as a transitional link between anamniotes and amniotes.

Steroids play a decisive role in sex differentiation of non-mammalian vertebrates. In this regard, the expression of the estrogen-producing enzyme, the aromatase, is temperature-sensitive in species where temperature can reverse sex differentiation, as in the newt *Pleurodeles waltl*. This species has been an interesting model for the study of vertebrate sex differentiation because this phenomenon displays a genetic regulation which can be prevailed by temperature [10,11]. In *Pleurodeles waltl*, sex determination shows female heterogamety: males have ZZ sex chromosomes and females have ZW sex chromosomes. At an ambient temperature (20 ± 2 °C), the sexual phenotype of *Pleurodeles waltl* conforms with its sex chromosome system. However, when the larvae are reared at 32 °C from stage 42 to stage 54 (thermosensitive period), the ZW animals are sex-reversed into functional males. At ambient temperatures, aromatase activity was detected at low levels in both ZZ and ZW larvae at stages 47 and 50, with stage 52 larvae having significantly higher levels of activity in females than in males. At higher temperatures, the activity remained low in the gonads of ZZ males, whereas it markedly increased in the gonads of ZW females. Exposure to 32 °C for 48 h significantly decreased the gonadal aromatase activity in ZW individuals [10]. These findings support the hypothesis that aromatase could be crucial for the temperature-sensitive sex-reversing effect on the gonads. Furthermore, in this species the gene encoding aromatase might be one of the master genes involved in the process leading to the differentiation of gonads [11].

Aromatase involvement in testicular differentiation is also hypothesized in *Rana rugosa*. ZZ embryos of this species treated with 17β-estradiol exhibited increasing aromatase expression after Day 21 and formed ovaries [12]. Androgen receptor (AR) antagonist treatment also induced high expression of aromatase and ovarian differentiation in these ZZ embryos. Furthermore, in males co-treated with an aromatase inhibitor and 17β-estradiol, the undifferentiated gonads developed into testes despite a high expression of aromatase. ZZ embryos co-treated with androgen and 17β-estradiol prior to and during an estrogen-sensitive period, respectively, developed testes too. In ZW females of this species, AR expression persisted at a low level, whereas aromatase expression increased after Day 18. Short-term treatment with an aromatase inhibitor was ineffective in preventing ovarian differentiation, whereas long-term treatment resulted in testes developing from the ovarian structure.

Both CYP19 mRNA expression, gene for P450 aromatase, and aromatase activity were low in the testes of *Xenopus laevis* adults. Forskolin, an inducer of aromatase, upregulated the CYP19 gene expression in testicular explants [13]. Furthermore, the CYP19 mRNA expression was detected in this species during ontogeny from the egg stage until metamorphosis, suggesting the involvement of aromatase in the sex differentiation of gonads [14].

However, the existing literature has few reports on the role of the expression/activity of aromatase and estrogens in adult amphibian testes, and most of such studies are restricted to anurans.

*Pelophylax esculentus* is a seasonal breeder [15,16,17,18,19,20,21,22,23]. The reproductive cycle of this species is controlled by the hypothalamus–pituitary–gonad axis, which regulates the release of sex steroids from gonads through a feedback mechanism [24,25]. The testis comprises germ cells forming clusters in the same stage of cytodifferentiation. Sertoli cells are present within the seminiferous tubules, and Leydig cells are present in the interstitial tissue among the seminiferous tubules. Spermatogenesis begins prior to the breeding season when the androgen levels suddenly increase. Testosterone levels were significantly higher in the reproductive period compared to the post-reproductive period. In the post-reproductive period, there is a shift in steroid biosynthesis, with the E_2_ levels higher than those of testosterone [19,26]. Accordingly, the CYP19 mRNA expression in testes changes in relation to the reproductive status, with the expression being the highest in the post-reproductive period [18,20]. The same pattern is observed in the gene expression of 17β-estradiol receptors in these two periods. In particular, the expression levels of ERα and ERβ are higher in post-reproductive frog testes [20]. Therefore, it has been proposed that the aromatase–estradiol system might be involved in the interruption of the reproductive processes as its activation in the post-reproductive period inhibited androgen biosynthesis and thus the development of sexual secondary characters. Furthermore, immunocytochemical analysis has revealed the presence of ERβ in the spermatogonia (SPG), spermatocytes (SPC), spermatids (SPT), and the interstitial tissue, suggesting that E_2_, via ERβ, induces the activity of Akt-1, a factor involved in proliferation, survival, and metabolism [27].

Interestingly, the aromatase enzyme is expressed in the brain of *P. esculentus*. Brain CYP19 expression showed sex dimorphism and varied among seasonal reproductive periods [18,20].

The South American toad *Rhinella arenarum* is a non-seasonal breeder with cystic testes and a continuous spermatogenic cycle. A peculiar aspect of this species is that aromatase is not expressed in its testes. The Bidder’s organ, a structure located at one pole of each testis, is proposed to be the main source of 17β-estradiol, as evidenced by the expression of aromatase enzyme and the in vitro production of 17β-estradiol in this structure [28].

Therefore, the findings support the hypothesis that in amphibians, aromatase could be crucial for temperature-sensitive sex differentiation of gonads during ontogeny. Further, the aromatase–estradiol system could be involved in the block of spermatogenesis necessary for the interruption of the reproductive phase in adults of seasonal breeders. The absence of aromatase expression in the testis of species showing a continuous reproductive cycle strongly supports this hypothesis.

## 3. Reptiles

Reptiles were the first amniotic terrestrial vertebrates. Their testes are composed of convoluted seminiferous tubules enveloped by connective tissue containing Leydig cells. In each of these tubules, both germ cells at different maturation stages and Sertoli cells are present. Sperm release from these tubules is promoted by myoid cells [29,30]. Furthermore, the entire male genital tract of reptiles, including the organization of the epididymis, is similar to that of the other amniotes [31].

Similar to other vertebrates, a direct relation between temperature and aromatase expression has been found in the sex determination of reptiles during development. Studies conducted in *Trachemys scripta* [32,33,34], *Chrysemys picta*, and *Apalone mutica* [35] have revealed different profiles of P450 aromatase expression in the gonads of embryos incubated at different temperatures. Eggs of *Alligator mississippiensis* incubated at male development temperature exhibited lower aromatase levels compared to the embryos of eggs incubated at the female development temperature [36]. In addition, eggs incubated at female-promoting temperature developed as males when treated with an aromatase inhibitor [37]. In adult alligators, the testicular level of P450 aromatase was approximately ten times lower than that in the brain, indicating that most circulating 17β-estradiol originates from extragonadal synthesis [38].

The Italian wall lizard *Podarcis sicula* is a seasonal breeder. In this species, high levels of estrogen are responsible for blocking spermatogenesis, whereas high levels of testosterone are responsible for the resumption of this process [7,39,40]. P450 aromatase has been detected in testicular somatic cells and germ cells of this species during the seasonal cycle, particularly in spermatids and spermatozoa, except in early autumnal resumption, when the enzyme was detected only in Leydig cells [41]. Both mRNA and protein expression of aromatase presented two peaks, one in summer and the other in winter stasis. The highest levels of P450 aromatase were consistent with the increase in the levels of 17β-estradiol, which is responsible for spermatogenesis blocking that is typical of this species. On the contrary, in autumnal resumption, the levels of P450 aromatase decreased, along with 17β-estradiol levels, whereas the testosterone concentration increased, which subsequently lead to the recovery of spermatogenesis. In spring resumption as well as in the reproductive phase, intermediate P450 aromatase levels, low titers of 17β-estradiol, and the highest concentrations of testosterone that induced the resumption of spermatogenesis were observed [41]. Interestingly, a relationship between aromatase and the local neuropeptides PACAP (pituitary adenylate cyclase-activating polypeptide) and VIP (vasoactive intestinal peptide) was observed in the testes of *Podarcis sicula* [42,43,44,45,46,47]. The aromatase expression in testes is regulated by cAMP through the interaction of promoter CRE (cAMP response element) with the transcription factor CREB (cAMP response element-binding protein) [48,49]. Experimental evidence indicates that the activation of the PACAP/VIP system could influence P450 aromatase expression by increasing the cytoplasmic levels of cAMP [50]. Similarly, another in vitro investigation demonstrated that treatment of testes with PACAP and VIP induced the release of estrogens [45]. Furthermore, P450 aromatase and neuropeptides are reportedly colocalized in both epithelium and connective tissue of the epididymis [51,52].

The importance of estrogens in male gametogenesis in lizards has been confirmed by the presence of estrogen receptors in its testis, in both germ cells and somatic cells [53]. In detail, all germ cells express the mRNAs of both ERα and ERβ during the reproductive period, whereas during the stasis period, these receptors are expressed in spermatogonia [54]. Furthermore, in *Podarcis sicula*, ERs are immunolocalized within the epithelium of the epididymis during all reproductive cycles [53].

In the testes of the turtle *Trachemys scripta*, aromatase immunolocalization changes in different phases of the reproductive cycle. During the non-reproductive period, the protein is present in the somatic cells, Leydig cells, and Sertoli cells, whereas, during the reproductive period, it is also detected within germ cells, particularly spermatids and spermatozoa [55]. Finally, in *Chrysemys picta*, *Trachemys scripta*, and *Chelonia mydas*, estrogen receptors have been detected in germ cells and somatic cells of the testes as well as in the epididymis [55,56,57]. In *Trachemys scripta*, immunopositivity for ER receptors was detected in the testes during both stasis and resumption periods, with ERα localized only within the Sertoli cells and ERβ present in both spermatogonia and Sertoli cells; both ERα and ERβ have also been detected in the epididymis [58].

Aromatase immunolocalization is also reported in the testes of the Chinese rat snake *Zaocys dhumnades*. In particular, the protein is present in both spermatids and spermatozoa within the testis as well as in the seminiferous spherules of the vas deferens, which in this species represent the alternative sites for spermiogenesis [59]. The aromatase-immunopositive germ cells in this species expressed estrogen receptors.

In conclusion, studies conducted in some reptiles suggest an involvement of aromatase enzyme in the sexual differentiation of the gonads. Similar to what is described in frogs, in adult lizard testis the higher aromatase and estrogen levels in the post-reproductive phase seems to be responsible for the blocking of spermatogenesis. On the contrary, in turtles the immunolocalization of aromatase in germ cells during the reproductive phase, but not in those during the non-reproductive phase, could suggest a predominant role of this enzyme for germ cell differentiation during reproduction.

## 4. Birds

In birds, testes exhibit the typical organization of amniotes [8,60]. Numerous studies conducted in chicken embryos have demonstrated an important role of P450 aromatase and estrogens in gonadal sex differentiation [61]. P450 aromatase mRNA expression first occurs on Day 6.5 of incubation in the female gonad, whereas the enzyme was not detected in the male gonad [62,63,64]. Accordingly, during the development, the estrogen receptor gene was temporarily expressed in the male gonad and was restricted to an early stage of development [63]. The administration of fadrozole, a non-steroidal aromatase inhibitor, to the chicken prior to sexual differentiation of the gonads induced sex reversal in females, which developed atypical testes, with few spermatozoa, linked to their oviducts [65,66]. Conversely, a feminization process was observed in the embryos of males treated with 17β-estradiol [67]. However, a recent study demonstrated that in transgenic male chickens overexpressing aromatase, even the high levels of circulating estrogen were insufficient for maintaining a female gonadal phenotype in adult birds [67]. Studies performed in gynandromorph chickens (a rare, naturally occurring phenomenon in which one side of the animal appears male and the other female) demonstrated that somatic sex differentiation in birds is cell-autonomous and independent of sexual hormones. Hence, the hormonal effects in birds affect the gonadal sex but not the somatic sex [68].

Estradiol levels and P450 aromatase expression have been investigated in adult quail *Coturnix coturnix* during the reproductive cycle [8]. In its seasonal cycle, the quail testis exhibits four distinct phases: a resting phase (at the end of summer), a recrudescence phase (in the fall), a proliferative phase (at the end of winter and the beginning of spring), and a regression phase (spring and summer). The elevated and maximum spermatogenic activity were observed in fall–winter (short-day period) and at the beginning of spring, respectively, while a lower activity was observed during spring and summer (long-day period). Since both 17β-estradiol and androgen levels are higher in the reproductive period compared to the non-reproductive period, it is hypothesized that estrogens and androgens act synergistically in the beginning (recovery) of spermatogenesis. Accordingly, in quail testis, P450 aromatase expression was higher in the reproductive period compared to that in the non-reproductive period. The strong P450 aromatase immunopositivity in the spermatids and spermatozoa suggests that the enzyme could be involved in the control of spermiogenesis [8]. In line with these data, in the testes of gander *Anser anser domesticus*, E_2_ levels were higher in the breeding stage than in the non-reproductive phase [69]. However, in this species, ER expression (ERα and ERβ) in the testis exhibited the opposite pattern of being relatively more abundant in the non-breeding stage.

Estrogen receptor immunopositivity is also reported in the epithelial cells of the efferent ductules, epididymal duct, and ductus deferens in immature and sexually mature Japanese quail *Coturnix japonica*, although increased ER expression may occur during maturation [70]. Furthermore, the gonads of *C. japonica* become sexually differentiated upon activation of ERα by endogenous estrogens. Excessive activation of ERα, and not ERβ, during embryonic development, may disrupt this process [71].

In roosters, one of the avian species extensively used for commercial purposes, P450 aromatase expression has been detected in the testis, prevalently in spermatocytes, spermatids, and spermatozoa, along with epididymal cells and non-ciliated cells of the proximal efferent ducts. Moreover, P450 aromatase activity has been detected in the spermatozoa isolated from epididymis and ductus deferens [72,73]. High levels of estrogen receptors were detected in mature testes, indicating that estrogens could be important for modulating testicular functions [74]. ERβ is the isoform prevalent in the testis, whereas ERα is prevalent in both epididymis and deferent duct [72].

An excessive amount of aromatase enzyme reduces the reproductive performance in aging roosters. Testosterone metabolism mediated by aromatase enzyme is one of the reasons for reduced testosterone and low fertility in aging roosters [75]. Experimental studies have demonstrated that the administration of aromatase inhibitors could enhance the reproductive performance of aged commercial broiler breeder roosters. Roosters orally treated with letrozole, an aromatase inhibitor, exhibited an increase in the diameter of the seminiferous tubules and a higher number of spermatozoa inside the seminiferous tubules and epididymis compared to control animals [76,77,78]. The effect of letrozole on spermatogenesis could be mediated by an altered serum testosterone/17β-estradiol ratio. Exemestane, a steroidal aromatase inhibitor, reportedly increased fertility in aging broiler breeder roosters; in particular, the roosters treated with 0.5 mg of exemestane exhibited a high percentage of sperm concentration, total motility, progressive motility, membrane integrity, viability, and mitochondrial activity [75].

Upregulation of both aromatase and ERα mRNA levels were detected in the testis, epididymis, and deferent ducts of domestic turkey *Meleagris gallopavo* with yellow semen compared to those with white semen [79]. Furthermore, in both the testis and epididymis of the turkeys with yellow semen, high expression of ER β and increased E_2_ levels were observed.

In zebra finches, estrogen is considered the normal masculinizing hormone as the developing females treated with estradiol reportedly develop a masculine song system [80,81,82,83,84]. High aromatase activity is reported in the brain, whereas testis presented undetectable enzymatic activity [85,86]. Therefore, functional estrogen necessary for the masculinization of the song system is most probably derived from the brain and supplied with substrate from the adrenals [81,85]. Accordingly, estrogens were reported to circulate at high levels in males after castration [85]. Male brown-headed cowbirds *Molothrus ater* exhibit moderate levels of aromatase activity in their testes, suggesting that the sites of estrogen production may vary among different species [87].

Therefore, the findings demonstrate that aromatase and estrogens play a key role in bird gonadal sex differentiation during ontogeny. In adult birds the role of the aromatase–estrogen system varies among species. Particularly, in quail it seems to act synergistically with androgens in the recovery of spermatogenesis during the reproductive phase whereas in rooster an excessive amount of testis aromatase enzyme reduces the reproductive performance.

## 5. Summary and Perspectives

**Figure 1:**
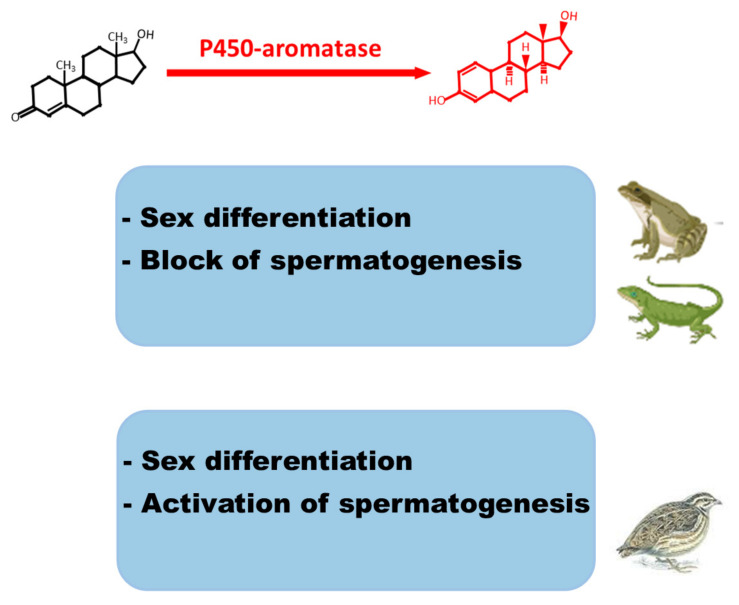
Schematic summary of testicular P450-aromatase/estradiol activities in Amphibians, Reptiles, and Birds. Block or activation of spermatogenesis refers to seasonal reproduction species.

**Figure 2:**
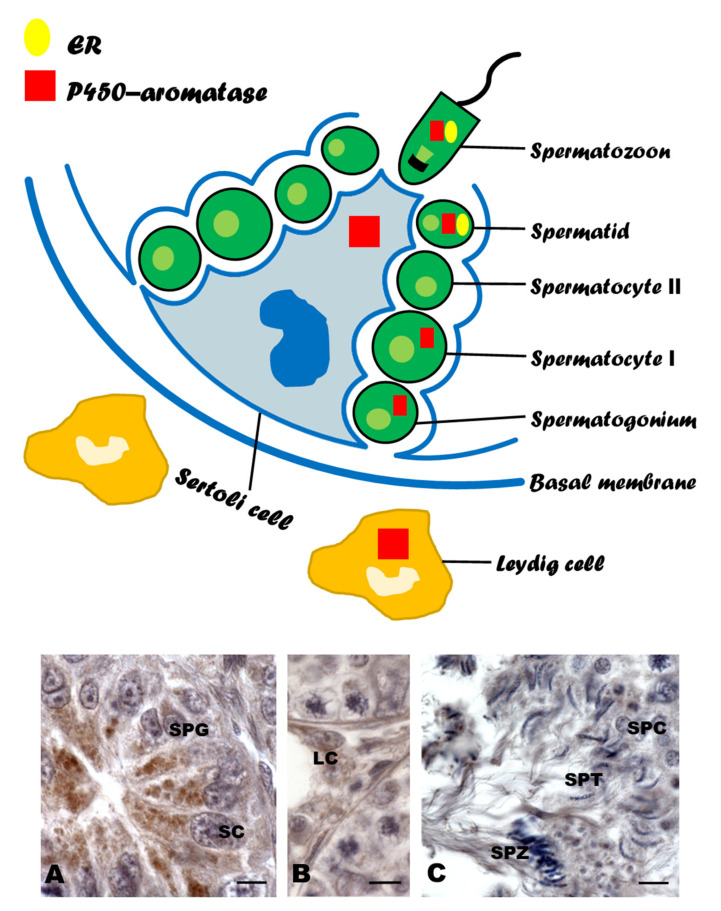
Schematic summary of estrogen receptor (ER) and P450 aromatase localizations in the testis of Amphibians, Reptiles and Birds. (**A**–**C**): Immunolocalization of P450 aromatase in *Podarcis sicula*. Signal has been localized: (**A**) spermatogonia (SPG) and Sertoli cells (SC); (**B**) Leydig cells (LC); (**C**) spermatocytes (SPC), spermatids (SPT), and spermatozoa (SPZ) (from [41]). Scale bars = 5 µm.

The aromatase–estrogen system appears to be crucial for the sex differentiation of females in amphibians, reptiles, and birds. Administration of aromatase inhibitors prior to sexual differentiation of the gonads results in the development of males rather than females. Therefore, aromatase has been postulated as the female sex determinant for species in which sex determination is temperature-dependent. CYP19 gene could be one such target for the temperature-sensitive sex-reversing effect on the gonads. Aromatase activity and estrogen levels play key roles in the reproductive cycle of the adults of seasonal breeding species. In *Pelophylax esculentus* and *Podarcis sicula*, the increased estrogen levels in the post-reproductive phase are responsible for the blocking of spermatogenesis, whereas, in the reproductive phase of *Coturnix*, estrogens together with testosterone promote spermatogenesis. Therefore, P450 aromatase serves as an “on/off” switch for spermatogenesis (Figure 1). Furthermore, the specific immunolocalization of aromatase and ERs in spermatids and testis/epididymal spermatozoa during the reproductive period suggests a prevalent role of estrogens in spermiogenesis and spermatozoa maturation (Figure 2).

The aromatase–estrogen system has been extensively investigated in roosters. An excessive amount of aromatase enzyme reduces the reproductive performance in roosters, whereas treatment with aromatase inhibitors may enhance fertility.

Finally, P450 aromatase has not been detected in the testes of male songbirds. The expression of estrogen-dependent masculine behaviors in these avian species have been demonstrated to be mediated by the synthesis of neuroactive estrogens in the brain from circulating androgens.

In conclusion, the specific role(s) that the aromatase–estrogen system may have in the male reproductive activity of non-mammalian species remains largely unknown. Data are available for only a few species. The studies indicate that the role of the aromatase–estrogen system in regulating testicular activity differs not only among the different classes of vertebrates but also among different species within the same class. Many questions remain unanswered, including: Why in some seasonally breeding species do estrogens seem to play a key role in the inhibition of spermatogenesis and in others, on the contrary, they have an important synergistic role with testosterone in promoting spermatogenesis? Which molecular mechanisms are involved in the modulation of aromatase synthesis/activity in the reproductive cycle? Do estrogens affect the sexual secondary characters of males? Finally, to clarify the role of estrogens in reproductive processes, future studies are needed to understand how estrogen receptors work in germ cells to regulate their differentiation. A better understanding of the reproductive biology of wild species in concert with knowledge of the environmental characteristics will undoubtedly help to identify putative external factors (i.e., temperature and photoperiod) involved in the regulation of the aromatase–estrogen system.

## Data Availability

The data presented in this study are available on request from the corresponding author.
